# Simultaneous detection and differentiation by multiplex real time RT-PCR of highly pathogenic avian influenza subtype H5N1 classic (clade 2.2.1 proper) and escape mutant (clade 2.2.1 variant) lineages in Egypt

**DOI:** 10.1186/1743-422X-7-260

**Published:** 2010-10-07

**Authors:** El-Sayed M Abdelwhab, Ahmed M Erfan, Christian Grund, Mario Ziller, Abdel-Satar Arafa, Martin Beer, Mona M Aly, Hafez M Hafez, Timm C Harder

**Affiliations:** 1 Friedrich-Loeffler-Institute, Greifswald-Insel Riems, Germany; 2 Animal Health Research Institute, Dokki, Giza, Egypt; 3 Institute of Poultry Diseases, Free University, Berlin, Germany

## Abstract

**Background:**

The endemic status of highly pathogenic avian influenza virus (HPAIV) of subtype H5N1 in Egypt continues to devastate the local poultry industry and poses a permanent threat for human health. Several genetically and antigenically distinct H5N1 lineages co-circulate in Egypt: Strains of clade 2.2.1 proper replicate mainly in backyard birds causing the bulk of human infections, while a variant lineage within 2.2.1 (2.2.1v) appears to be perpetuated mainly in commercial poultry farms in Egypt. Viruses of the 2.2.1v lineage represent drift variants escaping from conventional vaccine-induced immunity and some of these strains also escaped detection by commercial real time reverse transcriptase PCR (RT-qPCR) protocols due to mismatches in the primers/probe binding sites.

**Results:**

We developed therefore a versatile, sensitive and lineage-specific multiplex RT-qPCR for detection and typing of H5N1 viruses in Egypt. Analytical characterization was carried out using 50 Egyptian HPAIV H5N1 strains isolated since 2006 and 45 other avian influenza viruses (AIV). A detection limit of 400 cRNA copies per ml sample matrix was found. Higher diagnostic sensitivity of the multiplex assay in comparison to other generic H5 or M-gene based RT-qPCR assays were found by examination of 63 swab samples from experimentally infected chickens and 50 AIV-positive swab samples from different host species in the field in Egypt.

**Conclusions:**

The new multiplex RT-qPCR assay could be useful for rapid high-throughput monitoring for the presence of HPAIV H5N1 in commercial poultry in Egypt. It may also aid in prospective epidemiological studies to further delineate and better control spread of HPAIV H5N1 in Egypt.

## Background

The incursion of highly pathogenic avian influenza virus (HPAIV) of subtype H5N1 of phlyogenetic clade 2.2, subclade 2.2.1 [1], into Egypt in 2005/2006 caused severe economic losses in the commercial (previous total annual production of 850 million birds) and backyard sectors (250 million birds) of poultry production in this country [2]. The virus also possesses considerable zoonotic potential. Human cases of HPAIV H5N1 infection, characterized by a high fatality rate, started to occur due to virus exposure of humans at the poultry-human interface which is highly fissured in Egypt [3]. In order to restore poultry production capacities and to mitigate risks of an emergence of new virus variants with increased pandemic potential in the human population, efforts to control HPAIV H5N1 were given a high priority [4].

Despite intense control measures including blanket vaccination, surveillance and depopulation of infected poultry holdings, HPAI H5N1 has gained endemic status in Egyptian poultry populations [5] and continuous, year-round circulation of HPAI H5N1 virus has been reported [6-8]. This is at least in part due to the highly divergent evolution of H5N1 viruses in Egypt which seems to be accelerated and shaped by vaccine-induced selection pressure leading to the emergence of genetically and antigenically distinct viruses [9]. Strains of the parent subclade 2.2.1 proper (2.2.1p) are reported to circulate nationwide mainly in unvaccinated birds, particularly waterfowl from backyard holdings [7]. The vast majority of human infections (34 fatalities out of 109 infected cases until 22^nd ^June 2010 [10]) is attributable to viruses of this group. Since 2007, viruses of a variant sublineage emerged from clade 2.2.1. These viruses, which will here be referred to as lineage 2.2.1v, originated from and circulate predominantly in vaccinated commercial chickens [8]. These antigenically drifted strains were shown to escape immunity induced by standard H5 vaccination and are prevalent mainly in Lower Egypt, particularly in the Nile Delta [7,8,11,12]. Knowledge of the epidemiology, especially the transmission pathways, of those two lineages between commercial farms, backyard birds, feral birds and humans is incomplete but urgently required to improve control measures. So far, assignment of viruses to either lineage requires virus isolation and antigenic characterization by hemagglutination inhibition or sequencing and molecular analysis. No rapid typing tools are currently available.

A number of RT-qPCR assays for diagnosis and characterization with respect to subtype and pathogenicity of HPAIV H5N1 have been published. These assays target the matrix gene [13,14], the nucleoprotein gene [15,16], the neuraminidase and the hemagglutinin [17-23]. Egypt's surveillance program embarked on the H5-specific RT-qPCR assay [23], which is recommended by the World Organization of Animal Health (OIE). The assay was initially highly successful in detecting H5N1 infections in Egypt [5,7]. Since 2007, however, an increasing number of strains in Egypt escaping detection by this assay was reported [24]. Therefore, the aim of this study was to develop a sensitive multiplex RT-qPCR able to detect all HPAIV H5N1 variants of clade 2.2 currently circulating in Egypt and, simultaneously, to distinguish between conventional 2.2.1p strains and the 2.2.1v lineage of vaccine-driven escape variants.

## Results

A multiplex RT-qPCR assay was designed to detect HPAIV H5N1 viruses and simultaneously differentiate the main two major lineages circulating in Egypt; classic 2.2.1p viruses and the newly evolving clade 2.2.1v of antigenically drifted variant viruses. A single reaction assay using multiplexed primers and probes (three colours) was developed. Analytical characterization of the assay was carried out using 50 Egyptian HPAIV H5N1 strains and other avian pathogens. Diagnostic performance was examined with 63 swab samples from experimentally infected chickens and 50 avian influenza virus (AIV)-positive swab samples obtained from different host species in the field in Egypt.

### Real-Time RT-PCR optimization

**Figure 1:**
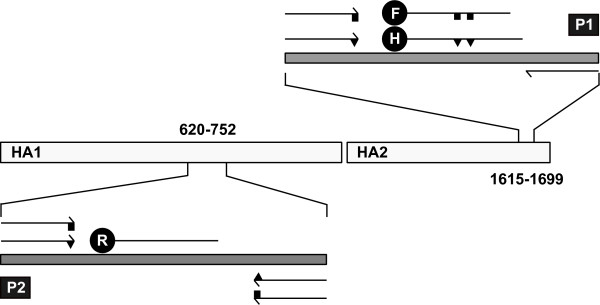
**Localization of primers and probes**. Localization of primers and probes as mentioned in table 1 along the HA gene is depicted. Two target regions (P1, P2) were chosen. Primers and probes were selected with specificity for the HA of Egyptian HPAIV H5N1 viruses either of lineage 2.2.1p (proper) or 2.2.1v (variant). Triangles (2.2.1p) and squares (2.2.1v) distinguish nucleotide positions specific for either lineage. Labelling of hydrolysis probes was with FAM (F, 2.2.1v), HEX (H, 2.2.1p) or ROX (2.2.1p+v).

Two strategies were combined to sensitively detect and distinguish viruses of clade 2.2.1p from 2.2.1v escape strains (Figure 1). Lineage-specific primers had disparate nucleotides at their most 3'-positions whereas lineage specific-probes were distinguished by two nucleotide positions towards the centre of the probe. Locked nucleotide chemistry was used to further increase the specificity of certain probes. Optimization runs were carried out using log_10 _dilution series of RNA extracted from A/chicken/Egypt/0879-NLQP/2008 (clade 2.2.1v), A/chicken/Egypt/NLQP-0918Q/2009 (clade 2.2.1p) and A/Whooper swan/Germany/R65/2006 (European clade 2.2) (data not shown). The concentration of primers and probes was optimized to increase the efficiency and sensitivity of amplification to final values shown in table 1. The lowest Ct values and highest ΔR_n _values for the multiplex RT-qPCR were observed using the following thermoprofile and the SuperScript III RT/Platinum *Taq *Mix chemistry: 30 min at 50°C and 2 min at 94°C, then 42 cycles of 94°C, 56°C and 68°C for 30 seconds each. No significant differences in sensitivity were evident when running the three RT-qPCRs separately indicating that the multiplex approach produced no relevant detrimental effects on amplification efficacy (data not shown).

**Table 1:** Oligonucleotide primers and probes designed for this study

**No**.	Primers/probes	Sequence 5'- 3'	Conc.^1 ^(nM)	Position^2^	Amplificate size (bp)
1	P1FW_2.2.1p	GAR TCA ATA GGA AYT TAC CAA ATA CT**G**	400	1615-1641	85
2	P1FW_2.2.1v	GAA TCA ATA GGA ACT TAC CAA ATA CTA T**C**	800	1615-1643	
3	P1RV_2.2.1	AGA CCA GCC ACC ATT GAT TGC	400	1699-1679	
4	PRO1.1_2.2.1v	FAM-ACA G*T*G GC***A ***AG***T ***TCC CT-BHQ-1	64	1654-1670	
5	PRO1.2_2.2.1p	HEX-ACA GTG GCG AGC TCC CTA GC-BHQ-1	64	1652-1675	

6	P2FW_2.2.1p	GGA TTC ACC ATC C**R**A ATG ATG**C**	1600	620-641	106
7	P2FW_2.2.1v	GGG ATT CAC CAT CCA AAT GAT G**A**	1600	619-641	
8	P2RV_2.2.1p	CCG TTT ACC TTA GAT CTA GTA GCT AT**T**	1600	752-726	
9	P2RV_2.2.1v	CCG TTT ACC TTA GAT CTA GT**R **GCT AT**C**	1600	752-726	
10	PRO2_2.2.1	ROX -TAC CTA TAT TTC CGT TGG GAC ATC AAC ACT AAA-BHQ-2	64	675-707	

**^1 ^**Nanomolar concentration of a 25 μl reaction.

**^2^**Position relative to the initiating codon of A/chicken/Egypt/06541-NLQP/2006 (H5N1), GenBank accession no. EU372946.1

Bold face nucleotides indicate mismatch positions between clade 2.2.1p proper and lineage 2.2.1v variant viruses. Italics indicate use of locked nucleic acid nucleotides.

The specificity of primers and probes for either or both of the 2.2.1 lineages is indicated with their designation.

### Analytical characteristics

**Figure 2:**
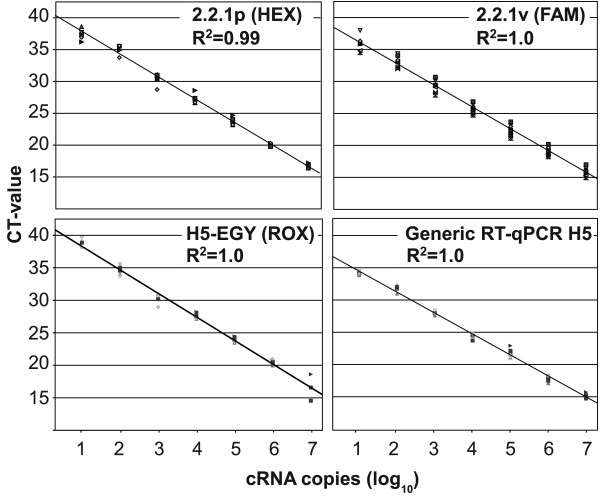
**Detection limits and amplification efficiency**. Detection limits and amplification efficiency of the multiplex RT-qPCR for detection and differentiation of clade 2.2.1p proper and 2.2.1v variant HPAIV H5N1 strains from Egypt using cRNA as a copy-based target. The standard curves were established by up to five independent runs. Average Ct values and variations are plotted against the cRNA copy numbers.

The current protocol was found to have a detection limit of approximately 2 - 5 RNA copies/reaction which amounts to 400 - 1000 copies per ml sample matrix when using cRNA as a copy-based standard. The dynamic ranges of target detection are summarized in figure 2.

A total of 50 HPAIV H5N1 isolates from Egypt obtained between 2006 and 2010 for which nucleotide sequences of the HA gene were available, were examined by the multiplex assay which assigned 33 of them to clade 2.2.1p (HEX-positive) whereas 15 isolates reacted like 2.2.1v strains (FAM-positive) (Additional file 1, Table S1). An exactly similar clustering was achieved for these strains in a phylogenetic analysis based on full length HA sequences (7, 8 and Additional file 1, Table S1). In addition, two isolates reacted positive for both 2.2.1p and 2.2.1v lineages. All 50 isolates were also detected by the ROX probe of the multiplex RT-qPCR (Additional file 1, Table S1). For 40 isolates a comparison with the generic H5 RT-qPCR validated by Slomka et al. [23] was performed showing markedly lower Ct values, corresponding to a higher sensitivity, for the ROX probe assay.

In addition, several, but not all, non-Egyptian H5 viruses could also be detected by the multiplex RT-qPCR with equal or slightly lower sensitivity compared to the generic H5 protocol described by Slomka et al. [23]. Negative results (no measurable Ct obtained [> > 40]) were generated with all non-H5 AI viruses as well as with other avian viral or bacterial pathogens (Additional file 2, Table S2).

### Diagnostic performance

**Figure 3:**
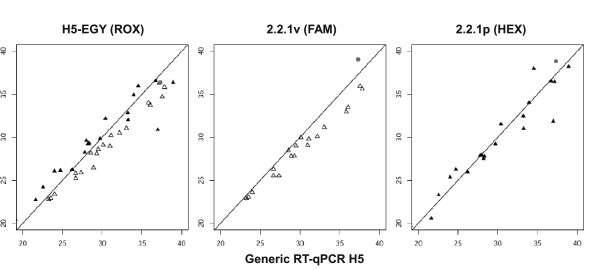
**Diagnostic performance characteristics (experimental infections)**. Diagnostic performance characteristics of a newly developed H5-specific multiplex RT-qPCR for detection and differentiation of clade 2.2.1p proper and 2.2.1v variant HPAIV H5N1 strains from Egypt compared to standard H5 RT-qPCR (Slomka et al. [20]). The analysis comprises swab samples obtained from SPF chickens after experimental infection with A/chicken/Egypt/0879-NLQP/2008 (clade 2.2.1v, open triangles, n = 34 samples) or A/chicken/Egypt/NLQP-0918Q/2009 (clade 2.2.1p, black triangles, n = 29 samples). See Additional file 3, Table S3, for individual values. Grey dots represent samples in which both clades were detected. X-axis: Ct-values obtained by the generic H5 RT-qPCR [20]. Y-axis: Ct-values generated by the specific component of the multiplex RT-qPCR as indicated above each graph.

Swab samples (n = 63) originating from SPF chickens which were experimentally infected with A/chicken/Egypt/0879-NLQP/2008 (clade 2.2.1v) or A/chicken/Egypt/NLQP-0918Q/2009 (clade 2.2.1p) were examined by the multiplex RT-qPCR and compared to Ct values obtained with an H5-specific assay described by Slomka et al. [23]. Results are shown in Additional file 3, Table S3, and Figure 3. Swabs collected from individual birds were selected on basis of Ct values obtained by a generic M gene-targeted RT-qPCR (14, not shown) so as to represent a wide range of samples with low to high concentrations of AIV RNA. Special emphasis was put on samples with Ct-values around 35. The multiplex assay assigned the correct lineage within clade 2.2.1 for all samples. One sample (#15, Additional file 3, Table S3) yielded a weak false-positive signal (Ct 39.03) in the 2.2.1v specific assay although the sample came from a chicken that was infected by a 2.2.1p proper virus. However, upon repeated RNA extraction and analysis of the sample, this false-positive reaction was not reproducible which indicates a possible spill-over contamination possibly during the previous extraction procedure. No significant differences in Ct values were evident between the 2.2.1p specific assay (HEX) of the multiplex RT-qPCR and the Slomka H5 RT-qPCRs (Figure 3a). However, both the 2.2.1v specific assay (FAM) and the new Egyptian H5.specific assay (ROX) of the multiplex mixture were significantly more sensitive (p = 0,027 and p ≤ 0,001) than the generic H5 RT-qPCR (Figures 3b-c).

**Figure 4:**
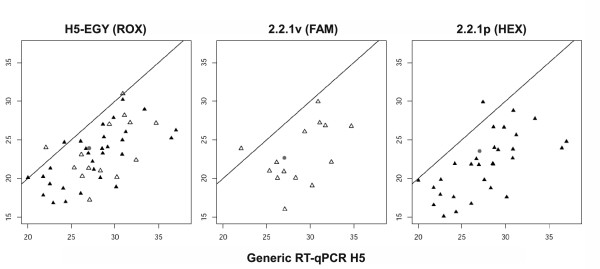
**Diagnostic performance characteristics (field samples)**. Diagnostic performance characteristics of a newly developed H5-specific multiplex RT-qPCR for detection and differentiation of clade 2.2.1p proper (black triangles) and 2.2.1v variant HPAIV H5N1 strains (open triangles) from Egypt compared to standard H5 RT-qPCR [20]. The analysis comprises 50 M-PCR positive field samples obtained within the frame of the Egyptian poultry surveillance program from commercial poultry farms (n = 11) and backyard holdings (n = 39). See Additional file 4, Table S4, for individual values. Grey dots represent samples in which both clades were detected. X-axis: Ct-values obtained by the generic H5 RT-qPCR [20]. Y-axis: Ct-values generated by the specific component of the multiplex RT-qPCR as indicated above each graph.

Oropharyngeal or cloacal swabs were also sampled in commercial farms (n = 11) and backyard poultry holdings (n = 39) in Egypt. A total of 50 samples pretested by M-specific RT-qPCR [14] to be positive for AIV was examined using the H5 multiplex RT-qPCR assay and the generic H5 protocol [23]. The multiplex RT-qPCR found all 50 samples positive for subtype H5 (ROX). Among them, 32 were assigned to clade 2.2.1p and 15 to lineage 2.2.1v (Additional file 4, Table S4, and figure 4) while three samples were positive for both. Only 45 samples tested positive in the generic H5 RT-qPCR using the original protocol by Slomka et al. [23], and on average the Ct values produced with the newly developed multiplex assay were significantly lower (p < < 0,001) than those obtained in both the M and the H5 generic RT-qPCRs (Figures 4a-c).

## Discussion

We report here the development of a multiplex RT-qPCR assay for detection and differentiation of Egyptian H5N1 HPAI viruses of clade 2.2.1 proper (p) and the emerging 2.2.1 lineage of viruses which escape standard vaccine-induced immunity (designated here 2.2.1v). The assay was shown to have a detection limit of 2-5 cRNA copies per reaction. Based on a phylogenetic analysis of 50 isolates tested [7,8], the assay is fully specific with regard to assigning the Egyptian H5N1 isolates to either phylogenetic cluster. No unspecific reactivity with either non-H5 AIV or other avian pathogens was evident. However, the assay can not be used for generic detection of subtype H5 viruses as a large percentage of non-Egyptian H5 subtype strains could not be detected.

In terms of diagnostic performance regarding the HPAIV H5N1 strains currently circulating in Egypt, the multiplex assay was at least equal to standard RT-qPCRs targeting the M gene of AIV and superior to a generic H5 RT-qPCR [23] when examining swabs which originated from experimental infections or from field samples of poultry holdings in Egypt. The generic H5-specific RT-qPCR assay described by Slomka et al. [23] apparently missed five Egyptian field samples, possibly due to mismatches in binding regions of primers and/or probes; one of the missed samples was assigned to lineage 2.2.1v while four of them belonged to 2.2.1p. In three field samples the multiplex assay detected both clades with almost similar Ct values (Additional file 4, Table S4, #9, #17, #18). The field samples analysed were derived from pooled swabs of five birds of each holding; as such it can not be excluded that infections with both clades occurred simultaneously at these holdings. Work is in progress to clarify these cases by sequencing clones of HA gene fragments. A similar situation was also encountered with two isolates (Additional file 1, Table S1, #3, #18); alignment of primer and probe sequences with the published sequences of these isolates, however, did not yield any hint for an unspecific reactivity. A contamination of these isolates can only be excluded by sequencing clones of HA gene fragments.

Reducing the amount of circulating HPAIV H5N1 virus by concerted actions of rapid and specific testing, culling and vaccination of poultry is the key to mitigate the risk of human infections and fatalities in Egypt. Controlling the endemic HPAIV H5N1 situation in Egypt is particularly painstaking because of [1] the concentration of the majority of commercial and backyard poultry business in a very small part of the whole country (Nile valley and, particularly, Nile delta), [2] the integration/contacts of backyard birds within small commercial poultry farms (farms with 5.000-20.000 birds represent circa 75% of the poultry production), [3] the marketing system (random uncontrolled movement of birds to/from live bird markets), and [4] day labourers at commercial farms usually raise backyard birds in their houses. In addition, continuing viral evolution which is even further accelerated and skewed by vaccination pressure remains a daily challenge for diagnostic measures which are at the root of all efforts to control the situation. Characterization of currently circulating strains and, if required, adaptation of amplification-based diagnostic tools, such as introduced here, is essential to improve the situation.

## Conclusions

The necessity to update commercial and generic H5-specific RT-qPCRs for the Egyptian situation has been stressed recently [24]. The current assay provides this update. The assay is tailored to suite the special Egyptian situation. Therefore, the multiplex assay is not recommended for use elsewhere, particularly in areas where non-clade 2.2 HPAIV H5N1 are prevalent. Also, should new lineages of HPAIV H5N1 be introduced into Egypt, such as the 2.3.2 subclade viruses which already escaped from Central and South-eastern Asia to South-eastern Europe earlier in 2010, the current assay will need updating again. In addition to detection of clade 2.2.1p H5 HPAIV the multiplex assay also allows the positive identification of the 2.2.1v lineage of vaccine escape mutants. This lineage probably evolved in commercial chicken farms where vaccination using standard LPAIV H5 strains was practiced. Recent studies have shown that new vaccines might be required to efficiently induce protective immunity against lineage 2.2.1v viruses in poultry [11]. The multiplex assay therefore may also be instrumental in decision-making regarding the type of vaccine to be used for the specific outbreak situation.

## Methods

### Reference viruses and bacteria

A panel of 50 HPAIV H5N1 strains isolated in SPF-chicken eggs in the National Laboratory for Quality Control on Poultry Production (NLQP) in Egypt was used for determination of the analytical specificity and sensitivity of the PCR assays (Additional file 1, Table S1). In addition 42 further avian influenza viruses of subtypes H1 [4], H2 [5], H5 [22], H6 [5], H7 [3], and H9 [3] from the repository of German National Reference Laboratory for Avian Influenza, Friedrich-Loeffler Institute, were analysed (Additional file 2, Table S2). Non-orthomyxoviruses and several bacterial species were used to further determine the specificity of the assay.

### Primer/probe design

A collection of 316 near full length H5 gene segment sequences of H5N1 viruses circulating in Egypt between 2006 and 2010 was retrieved from the public GenBank data base. Sequences were aligned using MUSCLE [25] and manually edited. Primers and probes design were selected from a variable region of the HA2 gene for detection of an 85 bp fragment and a more conserved region in the HA1 gene region for detection of a 106 bp fragment (Table 1, Figure 1). Primers P1FW-Standard-EGY, P1RV-EGY and probe PRO1a-Standard-EGY were used for detection of the clade 2.2.1p strains (HEX channel). Primers P1FW-Variant-EGY, P1RV-EGY and probe PRO-Variant-EGY (FAM channel) were used for detection of the clade 2.2.1v variant strains. Primers P2FW-Standard-EGY, P2FW-Variant-EGY, P2RV-Standard-EGY and P2RV-Variant-EGY and probe PRO2-EGY were used for detection of both lineages via the ROX channel.

### Real time RT-PCR optimization

The concentration of primers and probes was optimized in separate PCRs (two primers, one probe) and re-adjusted when combined in the multiplex RT-qPCR to increase the efficiency of amplification. Likewise, different annealing temperatures ranging from 50 to 60°C and different chemistries (SuperScript III One-Step RT-PCR system with Platinum *Taq *DNA polymerase [Invitrogen]; Quantitect One step kit; [Qiagen]) were evaluated. Reactions were carried out in a 25-μl volume on an MX3005P real time PCR machine (Stratagene).

### Quantitative analysis

For preparation of standard controls, the cloned H5 gene segment from A/chicken/Egypt/0879-NLQP/2008 (clade 2.2.1v) and A/chicken/Egypt/NLQP-0918Q/2009 (clade 2.2.1p) was used for generating cRNA in vitro by run-off transcriptions performed as previously described [18]. Detection limit of the RT-qPCR was determined using 10-fold serial dilutions (10^1^-10^7 ^copies) of cRNA.

### Samples from experimentally infected chickens

All animal experiments were conducted following official German animal welfare regulations (LALLF M-V/TSD/7221.3-2.1-031/09). Six weeks old SPF chickens (n = 10, each) were infected by the oculo-nasal route with a dose of 10^6.0 ^TCID_50 _of A/chicken/Egypt/0879-NLQP/2008 (clade 2.2.1v) or A/chicken/Egypt/NLQP-0918Q/2009 (clade 2.2.1p) (Grund et al., unpublished). RNA was extracted from mixed oropharyngeal and cloacal swabs collected from individual birds 2 or 7 days post infection (dpi). RNA was used in quantitative RT-qPCRs described in this study and compared to M and H5 based RT-qPCR assays as previously described [14,23].

### Field samples

Tracheal and cloacal swabs were collected both from poultry at commercial farms (n = 11) and backyard holdings (n = 39) in the frame of the national surveillance scheme in poultry sectors in Egypt from 2008 to 2010. Oropharyngeal and cloacal swabs from five birds were pooled for RNA extraction and PCR analysis.

### Nucleic acid extraction

Extraction of RNA from 140 μl of allantoic fluid (RNA viral isolates) or swab fluid (field samples, experimental infections) was carried out using the QIAamp viral RNA mini kit (Qiagen, Hilden, Germany) following the manufacturer's instructions. The RNA was eluted from the columns with 50 μl of DEPC-treated water and used immediately or after storage at -80°C. Likewise, DNA was extracted from bacterial species and DNA viruses (Additional file 2, Table S2) using the QIAamp DNA mini kit (Qiagen, Hilden, Germany).

### Statistics

In addition to the descriptive evaluation of the test results, the technical sensitivity of the new multiplex RT-qPCR was investigated by comparing positive Ct-values with those of the standard H5-specific RT-qPCR test recommended by Slomka et al. [23]. For this purpose, Fisher's exact tests were applied considering the one-sided hypothesis of achieving lower Ct-values by the multiplex RT-qPCR than by the standard test. All statistical calculations have been performed using R, Version 2.8.1 (2008-12-22) [26].

## List of abbreviations

AIV: avian influenza virus; DPI: days post infection; FLI: Friedrich-Loeffler-Institute; HPAI: highly pathogenic avian influenza; LPAIV: avian influenza virus of low pathogenicity; HA: hemagglutinin; NLQP: National Laboratory for Quality Control on Poultry Production; O.I.E.: World Organization for Animal Health; RT-qPCR: real time reverse transcriptase polymerase chain reaction.

## Competing interests

The authors declare that they have no competing interests.

## Authors' contributions

ESAW carried out part of the development studies, the analytical and the diagnostic evaluation; he performed the sequence alignments and helped to draft the manuscript. AME and ASA carried out parts of the analytical and diagnostic evaluation using Egyptian samples. MZ carried out the statistical tests. CG, MMA and HMH provided samples for analysis, participated in the design of the study and helped to draft the manuscript. TCH conceived and coordinated the study, and drafted the manuscript. All authors read and approved the final manuscript.

## Supplementary Material

Additional file 1 **Detection and differentiation of HPAIV H5N1 isolates collected from commercial poultry and backyard birds in Egypt in 2006- 2010 by multiplex H5 RT-qPCR and a generic H5-specific RT-qPCR **[20]. Click here for file

Additional file 2 **Analytical specificity of the multiplex RT-qPCR for Egyptian HPAIV H5N1 using different avian influenza virus isolates and other avian pathogens**. Click here for file

Additional file 3 **Detection and differentiation of HPAIV H5N1 in selected swab samples collected from SPF chickens experimentally infected with Egyptian clade 2.2.1p proper or 2.2.1v variant HPAI H5N1 virus strains by multiplex H5 RT-qPCR compared to a standard generic H5 RT-qPCR protocol**. Click here for file

Additional file 4 **Detection and differentiation of HPAIV H5N1 in pooled swab samples collected from commercial poultry and backyard birds in Egypt in 2008- 2010 by multiplex H5 RT-qPCR compared to standard generic H5 and M gene RT-qPCR protocols**. Click here for file

## References

[B1] WHO/OIE/FAO H5N1 Evolution Working Group Continuing progress towards a unified nomenclature for the highly pathogenic H5N1 avian influenza viruses: divergence of clade 2.2 viruses Influenza Other Respi Viruses 2009 3 59 62 10.1111/j.1750-2659.2009.00078.x 19496842 PMC4634523

[B2] Meleigy M Egypt battles with avian influenza Lancet 2007 370 553 554 10.1016/S0140-6736(07)61274-4 17710749

[B3] Fasina FO Ifende VI Ajibade AA Avian influenza A(H5N1) in humans: lessons from Egypt 2009 15 4 Euro Surveill http://www.eurosurveillance.org/ViewArticle.aspx?ArticleId = 19473 pii = 19473 20122384

[B4] Peiris JS de Jong MD Guan Y Avian influenza virus (H5N1): a threat to human health Clinical Microbiol Rev 2007 20 243 267 10.1128/CMR.00037-06 PMC1865597 17428885

[B5] Aly MM Arafa A Hassan MK Epidemiological findings of outbreaks of disease caused by highly pathogenic H5N1 avian influenza virus in poultry in Egypt during 2006 Avian Dis 2008 52 269 277 10.1637/8166-103007-Reg.1 18646456

[B6] Abdelwhab EM Selim A Arafa A Galal S Kilany WH Hassan MK Aly MM Hafez HM Circulation of avian influenza H5N1 in live bird markets in Egypt Avian Dis 2010 54 911 914 10.1637/9099-100809-RESNOTE.1 20608538

[B7] Arafa A Suarez DL Hassan MK Aly MM Phylogenetic analysis of HA and NA genes of HPAI-H5N1 Egyptian strains isolated from 2006 to 2008 indicates heterogeneity with multiple distinct sublineages Avian Dis 2010 54 345 349 10.1637/8927-051509-ResNote.1 20521657

[B8] Balish AL Davis T Saad MD El-Sayed N Esmat H Tjaden JA Earhart KC Ahmed LE Abd El-Halem M Hakem M Ali AM Nassif SA El-Ebiary EA Taha M Aly MM Arafa A O'Neill E Xiyan X Cox NJ Donis RO Klimov AI Antigenic and genetic diversity of highly pathogenic avian influenza A (H5N1) viruses isolated in Egypt Avian Dis 2010 54 329 334 10.1637/8903-042909-Reg.1 20521654

[B9] van den Berg T Lambrecht B Marché S Steensels M Van Borm S Bublot M Influenza vaccines and vaccination strategies in birds Comp Immunol Microbiol Infect Dis 2008 31 121 165 10.1016/j.cimid.2007.07.004 17889937

[B10] WHO. 2010. World Health Organization Situation updates--avian influenza http://www.who.int/csr/disease/avian_influenza/updates/en/index.html [cited 2010, June 13;]

[B11] Kim J Kayali G Walker D Forrest HL Ellebedy AH Griffin YS Rubrum A Bahgat MM Kutkatd MA Ali MAA Aldridge JR Negovetich NJ Krauss S Webby RJ Webster RG Puzzling inefficiency of H5N1 influenza vaccines in Egyptian poultry Proc Natl Acad Sci 2010 107 11044 9 10.1073/pnas.1006419107 20534457 PMC2890765

[B12] Terregino C Toffan A Cilloni F Monne I Bertoli E Castellanos L Amarin N Mancin M Capua I Evaluation of the protection induced by avian influenza vaccines containing a 1994 Mexican H5N2 LPAI seed strain against a 2008 Egyptian H5N1 HPAI virus belonging to clade 2.2.1 by means of serological and in vivo tests Avian Pathol 2010 39 215 222 10.1080/03079451003781858 20544428

[B13] Di Trani L Bedini B Donatelli I Campitelli L Chiappini B De Marco MA Delogu M Buonavoglia C Vaccari GA Sensitive one-step real-time PCR for detection of avian influenza viruses using a MGB probe and an internal positive control BMC Infect Dis 2006 6 87 10.1186/1471-2334-6-87 16725022 PMC1524785

[B14] Spackman E Senne DA Myers TJ Bulaga LL Garber LP Perdue ML Lohman K Daum LT Suarez DL Development of a real-time reverse transcriptase PCR assay for type A influenza virus and the avian H5 and H7 haemagglutinin subtypes J Clin Microbiol 2002 40 3256 3260 10.1128/JCM.40.9.3256-3260.2002 12202562 PMC130722

[B15] Muradrasoli S Mohamed N Belák S Czifra G Herrmann B Berencsi G Blomberg J Broadly targeted triplex real-time PCR detection of influenza A, B and C viruses based on the nucleoprotein gene and a novel "MegaBeacon" probe strategy J Virol Methods 2010 163 313 322 10.1016/j.jviromet.2009.10.017 19879296 PMC7172653

[B16] Suarez DL Das A Ellis E Review of rapid molecular diagnostic tools for avian influenza virus Avian Dis 2007 51 201 208 10.1637/7732-101006-REGR.1 17494554

[B17] Chen W He B Li C Zhang X Wu W Yin X Fan B Fan X Wang J Real-time RT-PCR for H5N1 avian influenza A virus detection J Med Microbiol 2007 5 603 607 10.1099/jmm.0.47014-0 17446281

[B18] Hoffman E Stech J Guan Y Webster RG Perez D Universal primer set for the full-length amplification of all influenza A viruses Arch Virol 2001 146 2275 2289 10.1007/s007050170002 11811679

[B19] Li PQ Zhang J Muller CP Chen JX Yang ZF Zhang R Li J He YS Development of a multiplex real-time polymerase chain reaction for the detection of influenza virus type A including H5 and H9 subtypes Diagn Microbiol Infect Dis 2008 61 192 197 10.1016/j.diagmicrobio.2008.01.007 18296004

[B20] Lu YY Yan JY Feng Y Xu CP Shi W Mao HY Rapid detection of H5 avian influenza virus by TaqMan-MGB real-time RT-PCR Lett Appl Microbiol 2008 46 20 25 17944840 10.1111/j.1472-765X.2007.02253.x PMC7197896

[B21] Ng LF Barr I Nguyen I Noor SM Tan RS Agathe LV Gupta S Khalil H To TL Hassan SS Ren EC Specific detection of H5N1 avian influenza A virus in field specimens by a one-step RT-PCR assay BMC Infect Dis 2006 6 40 10.1186/1471-2334-6-40 16512903 PMC1421410

[B22] Payungporn S Chutinimitkul S Chaisingh A Damrongwantanapokin D Buranathai C Amonsin A Theamboonlers A Poovorawan YJ Single step multiplex real-time RT-PCR for H5N1 influenza A virus detection J Virol Methods 2006 131 143 147 10.1016/j.jviromet.2005.08.004 16183140

[B23] Slomka MJ Pavlidis T Banks J Shell W McNally A Essen S Brown IH Validated H5 Eurasian real-time reverse transcriptase-polymerase chain reaction and its application in H5N1 outbreaks in 2005-2006 Avian Dis 2007 51 373 377 10.1637/7664-060906R1.1 17494587

[B24] Arafa A Selim AA Hassan MK Aly MM Genetic characterization of variant strains of highly pathogenic avian influenza H5N1 that escaped detection by real-time reverse transcriptase-PCR diagnostic tests Avian Dis 2010 54 673 676 10.1637/8720-032109-ResNote.1 20521713

[B25] Edgar RC MUSCLE: multiple sequence alignment with high accuracy and high throughput Nucleic Acids Res 2004 32 1792 1797 10.1093/nar/gkh340 15034147 PMC390337

[B26] R Development Core Team R: A language and environment for statistical computing R Foundation for Statistical Computing, Vienna, Austria 2008 http://www.R-project.org ISBN 3-900051-07-0

