# Supplementary material for: Simultaneous detection and differentiation by multiplex real time RT-PCR of highly pathogenic avian influenza subtype H5N1 classic (clade 2.2.1 proper) and escape mutant (clade 2.2.1 variant) lineages in Egypt
Source: Virol J. 2010 Oct 7;7:260. doi: 10.1186/1743-422X-7-260 (PMC2958913; doi:10.1186/1743-422X-7-260)
Supplement: Additional file 1 — Detection and differentiation of HPAIV H5N1 isolates collected from commercial poultry and backyard birds in Egypt in 2006- 2010 by multiplex H5 RT-qPCR and a generic H5-specific RT-qPCR [20]. [file 1743-422X-7-260-S1.DOC]

**Table S1.** Detection and differentiation of HPAIV H5N1 isolates collected from commercial poultry and backyard birds in Egypt in 2006- 2010 by multiplex H5 RT-qPCR and a generic H5-specific RT-qPCR (20).

| **No.** | **Host** | **Year** | **Accession No.** | **Multiplex RT-qPCR assay** | | | **Generic**  **RT-qPCR H5** |
| --- | --- | --- | --- | --- | --- | --- | --- |
|  |  |  |  | **2.2.1 proper HEX** | **2.2.1 variant**  **FAM** | **2.2.1 all**  **ROX** |  |
| 1 | Chicken | 2006 | EU372947 | 6^1^ | Neg | 7 | 11.01 |
| 2 | Chicken | 2006 | EU372944 | 15.05 | Neg | 16.02 | 15.05 |
| 3 | Chicken | 2006 | EU372946 | 21.68 | 21.65 | 22.29 | 27.62 |
| 4 | Chicken | 2007 | GQ184215 | 8.68 | Neg | 9.88 | 13.7 |
| 5 | Chicken | 2007 | EU496389 | Neg | 7.05 | 8.11 | 12.58 |
| 6 | Chicken | 2007 | EU496396 | Neg | 9.86 | 11.14 | 16.91 |
| 7 | Chicken | 2007 | EU496388 | Neg | 13.86 | 15 | 19.62 |
| 8 | Duck | 2007 | EU496392 | 7.99 | Neg | 9.11 | 13.55 |
| 9 | Duck | 2007 | EU496391 | 9.05 | Neg | 9.38 | 19.14 |
| 10 | Turkey | 2007 | EU496390 | Neg | 17.99 | 19.37 | 25.08 |
| 11 | Chicken | 2008 | GU811713 | 8.87 | Neg | 10.11 | 13.65 |
| 12 | Chicken | 2008 | GQ184222 | 9.18 | Neg | 10.46 | 14.35 |
| 13 | Chicken | 2008 | CY041298 | 8.1 | Neg | 9 | 15.93 |
| 14 | Chicken | 2008 | GQ184220 | 7.81 | Neg | 8.29 | 12.06 |
| 15 | Chicken | 2008 | GQ184249 | 9.9 | Neg | 11.07 | 12.98 |
| 16 | Chicken | 2008 | GQ184224 | Neg | 8.01 | 9 | 14.09 |
| 17 | Chicken | 2008 | GQ184247 | Neg | 7.3 | 7.56 | 12.21 |
| 18 | Chicken | 2008 | GU811714 | 21.87 | 24.24 | 21.76 | 27.52 |
| 19 | Chicken | 2008 | Pending | Neg | 22.65 | 30 | n.d. |
| 20 | Chicken | 2008 | Pending | Neg | 28.04 | 28.46 | n.d. |
| 21 | Chicken | 2008 | Pending | Neg | 28.44 | 28.26 | n.d. |
| 22 | Chicken | 2008 | GQ184238 | Neg | 25.82 | 29.36 | n.d. |
| 23 | Chicken | 2008 | GQ184221 | Neg | 28.27 | 30.93 | n.d. |
| 24 | Chicken | 2008 | Pending | 25.43 | Neg | 30.15 | n.d. |
| 25 | Chicken | 2008 | Pending | Neg | 23.36 | 26.44 | n.d. |
| 26 | Duck | 2008 | GQ184235 | 11.95 | Neg | 12.45 | 17.36 |
| 27 | Duck | 2008 | GQ184237 | 8.83 | Neg | 9.38 | 12.35 |
| 28 | Duck | 2008 | Pending | 28.49 | Neg | 34.35 | n.d. |
| 29 | Duck | 2008 | Pending | 31.46 | Neg | 29.05 | n.d. |
| 30 | Chicken | 2009 | Pending | Neg | 9.03 | 9.64 | 15.44 |
| 31 | Chicken | 2009 | GU002695 | Neg | 10.96 | 12.09 | 17.09 |
| 32 | Chicken | 2009 | GU002705 | Neg | 8.98 | 10.16 | 14.08 |
| 33 | Chicken | 2009 | Pending | 28.28 | Neg | 26.29 | n.d. |
| 34 | Duck | 2009 | GU002696 | 8.96 | Neg | 10.28 | 16.14 |
| 35 | Duck | 2009 | GU002697 | 8.99 | Neg | 9.1 | 13.68 |
| 36 | Duck | 2009 | GU002673 | 7.5 | Neg | 8.12 | 13.95 |
| 37 | Duck | 2009 | Pending | 7.7 | Neg | 8.18 | 12.75 |
| 38 | Duck | 2009 | GU002675 | 7.5 | Neg | 8.16 | 12.99 |
| 39 | Duck | 2009 | GU002681 | 7.3 | Neg | 8.15 | 12.95 |
| 40 | Duck | 2009 | Pending | 10.81 | Neg | 12.16 | 17.58 |
| 41 | Duck | 2009 | GU002686 | Neg | 6.9 | 8.17 | 13.05 |
| 42 | Goose | 2009 | GU002677 | 9.04 | Neg | 10.32 | 15.83 |
| 43 | Turkey | 2009 | GU002702 | Neg | 8.09 | 9.26 | 14.25 |
| 44 | Chicken | 2010 | Pending | 10.75 | Neg | 11.93 | 15.96 |
| 45 | Chicken | 2010 | Pending | 12.93 | Neg | 16.5 | 20.22 |
| 46 | Chicken | 2010 | Pending | 10.86 | Neg | 11.97 | 23.76 |
| 47 | Chicken | 2010 | Pending | 7.95 | Neg | 8.89 | 15.76 |
| 48 | Chicken | 2010 | Pending | Neg | 10.79 | 10.47 | 14.79 |
| 49 | Chicken | 2010 | Pending | Neg | 10.85 | 10.31 | 22.14 |
| 50 | Chicken | 2010 | Pending | Neg | 8.99 | 8.64 | 16.42 |
