# Supplementary material for: Simultaneous detection and differentiation by multiplex real time RT-PCR of highly pathogenic avian influenza subtype H5N1 classic (clade 2.2.1 proper) and escape mutant (clade 2.2.1 variant) lineages in Egypt
Source: Virol J. 2010 Oct 7;7:260. doi: 10.1186/1743-422X-7-260 (PMC2958913; doi:10.1186/1743-422X-7-260)
Supplement: Additional file 2 — Analytical specificity of the multiplex RT-qPCR for Egyptian HPAIV H5N1 using different avian influenza virus isolates and other avian pathogens. [file 1743-422X-7-260-S2.DOC]

**Table S2.** Analytical specificity of the multiplex RT-qPCR for Egyptian HPAIV H5N1 using different avian influenza virus isolates and other avian pathogens.

|  | **Isolate** | **HA** | **NA** | **Multiplex RT-qPCR assay** | | | **Generic RT-qPCR H5** |
| --- | --- | --- | --- | --- | --- | --- | --- |
|  |  |  |  | **2.2.1 proper HEX** | **2.2.1 variant**  **FAM** | **2.2.1**  **all**  **ROX** |  |
| 1. 1 | A/ck/Scotland/59 | H5 | N1 | Neg | 36.69 | 34.14 | 27.8 |
|  | A/ck/Indonesien/R132/03 (clade 2.1) | H5 | N1 | 30.88 | 28.18 | 21.31 | 26.64 |
|  | A/ck/Indonesien/R60/05 (clade 2.1) | H5 | N1 | 34.1 | 30.85 | Neg | 28.05 |
|  | A/ck/GXLA/12/2004 (clade 1) | H5 | N1 | 30.35 | 27.93 | Neg | 28.09 |
|  | A/dk/Vietnam/TG24-01/05 (clade 1) | H5 | N1 | Neg | Neg | Neg | 31.58 |
|  | A/Cygnus cygnus/Germany/R65/2006 (clade 2.2) | H5 | N1 | 30.11 | Neg | Neg | 37.5 |
|  | A/Mergus albellus/Slovakia/Vh212_  R53706/06 (clade 2.2) | H5 | N1 | Neg | Neg | Neg | 37.45 |
|  | A/Black-necked grebe/Germany/  R1493/07 (clade 2.2) | H5 | N1 | 24.41 | Neg | Neg | 25.76 |
|  | A/dk/Germany/R1959/07 (clade 2.2) | H5 | N1 | Neg | Neg | Neg | 27.29 |
|  | A/tk/Poland/36/08 (clade 2.2) | H5 | N1 | 26.99 | Neg | Neg | 27.54 |
|  | A/Common teal/Germany/Wv632/05 | H5 | N1 | 31.55 | 28.8 | 34.92 | 22.59 |
|  | A/dk/Potsdam/1402/86 | H5 | N2 | 38.72 | Neg | Neg | 34.05 |
|  | A/ck/Italy/8/98 | H5 | N2 | 26.53 | 32.26 | Neg | 26.51 |
|  | A/dk/British Columbia/26-6/2005 | H5 | N2 | Neg | Neg | Neg | 31.65 |
|  | A/gs/Manitoba/428/2006 | H5 | N2 | 38.78 | Neg | 37.25 | 30.87 |
|  | A/Tern/South Africa/61 | H5 | N3 | Neg | 35.39 | Neg | 28.22 |
|  | A/Ostrich/Germany/R5-10/06 | H5 | N3 | 38.57 | 35.79 | Neg | 29.1 |
|  | A/tk/Germany/R1550/08 | H5 | N3 | Neg | 39.91 | Neg | 27.69 |
|  | A/dk/Potsdam/2216/84 | H5 | N6 | Neg | Neg | Neg | 24.15 |
|  | A/ck/Italy/22/98 | H5 | N9 | Neg | Neg | Neg | 30.66 |
|  | A/Mallard/Alberta/329/2006 | H5 | N9 | Neg | Neg | Neg | 33.27 |
|  | A/Mallard/British Columbia/544/2005 | H5 | N9 | 38.8 | Neg | Neg | 34.84 |
|  |  |  |  |  |  |  |  |
|  | A/RP/14/07 | H1 | N1 | Neg | Neg | Neg | Neg |
|  | A/Wild duck/Germany/R30/06 | H1 | N1 | Neg | Neg | Neg | Neg |
|  | A/Mallard/Germany/R4/08 | H1 | N1 | Neg | Neg | Neg | Neg |
|  | A/White fronted goose/Germany/R482/09 | H1 | N1 | Neg | Neg | Neg | Neg |
|  | A/Mallard duck/Germany/R2711/07 | H2 | N1 | Neg | Neg | Neg | Neg |
|  | A/Guinea fowl/Germany/DZ3/85 | H2 | N2 | Neg | Neg | Neg | Neg |
|  | A/Mute swan/Germany/R3227/07 | H2 | N3 | Neg | Neg | Neg | Neg |
|  | A/Mallard/Germany/SRa517K/07 | H2 | N5 | Neg | Neg | Neg | Neg |
|  | A/Greylag goose/Germany/R1487/08 | H2 | N9 | Neg | Neg | Neg | Neg |
|  | A/tk/Gvulot Israel/2009 | H6 | N1 | Neg | Neg | Neg | Neg |
|  | A/tk/Mass/3740/65 | H6 | N2 | Neg | Neg | Neg | Neg |
|  | A/tk/Germany/R617/07 | H6 | N2 | Neg | Neg | Neg | Neg |
|  | A/Mallard/Germany/Sum156/07 | H6 | N5 | Neg | Neg | Neg | Neg |
|  | A/Red-brested goose/Germany/R1/06 | H6 | N8 | Neg | Neg | Neg | Neg |
|  | A/Mute swan/Germany/R901/06 | H7 | N1 | Neg | Neg | Neg | Neg |
|  | A/Mute swan/Potsdam/62/81 | H7 | N3 | Neg | Neg | Neg | Neg |
|  | A/tk/Germany/R11/01 | H7 | N7 | Neg | Neg | Neg | Neg |
|  | A/tk/Wisconsin/66 | H9 | N2 | Neg | Neg | Neg | Neg |
|  | A/ck/Germany/45/98 | H9 | N2 | Neg | Neg | Neg | Neg |
|  | A/ck/Iran/R64/02 | H9 | N2 | Neg | Neg | Neg | Neg |
|  |  |  |  |  |  |  |  |
|  | Avian pneumovirus | - | - | Neg | Neg | Neg | Neg |
|  | Infectious bronchitis virus | - | - | Neg | Neg | Neg | Neg |
|  | Infectious laryngotracheitis virus | - | - | Neg | Neg | Neg | Neg |
|  | Newcastle Disease virus | - | - | Neg | Neg | Neg | Neg |
|  | Avian reovirus | - | - | Neg | Neg | Neg | Neg |
|  |  |  |  |  |  |  |  |
|  | *Mycoplasma gallisepticum* | - | - | Neg | Neg | Neg | Neg |
|  | *Mycoplasma synoviae* | - | - | Neg | Neg | Neg | Neg |
|  | *Staphylococcus aureus* | - | - | Neg | Neg | Neg | Neg |
|  | *Clostridium perfringens* | - | - | Neg | Neg | Neg | Neg |
|  | *Escherichia coli* | - | - | Neg | Neg | Neg | Neg |
|  | *Pasteurella multicida* serotype A | - | - | Neg | Neg | Neg | Neg |
