# Supplementary material for: Simultaneous detection and differentiation by multiplex real time RT-PCR of highly pathogenic avian influenza subtype H5N1 classic (clade 2.2.1 proper) and escape mutant (clade 2.2.1 variant) lineages in Egypt
Source: Virol J. 2010 Oct 7;7:260. doi: 10.1186/1743-422X-7-260 (PMC2958913; doi:10.1186/1743-422X-7-260)
Supplement: Additional file 3 — Detection and differentiation of HPAIV H5N1 in selected swab samples collected from SPF chickens experimentally infected with Egyptian clade 2.2.1p proper or 2.2.1v variant HPAI H5N1 virus strains by multiplex H5 RT-qPCR compared to a standard generic H5 RT-qPCR protocol. [file 1743-422X-7-260-S3.DOC]

**Table S3.** Detection and differentiation of HPAIV H5N1 in selected swab samples collected from SPF chickens experimentally infected with Egyptian clade 2.2.1 proper or 2.2.1 variant HPAI H5N1 virus strains by multiplex H5 RT-qPCR compared to a standard generic H5 RT-qPCR protocol.

| **No.** | **Day pi** | **Chicken no.** | **Lineage** | **Multiplex RT-qPCR assay** | | | **Generic**  **RT-qPCR H5** |
| --- | --- | --- | --- | --- | --- | --- | --- |
|  |  |  |  | **2.2.1 proper HEX** | **2.2.1 variant**  **FAM** | **2.2.1**  **all**  **ROX** |  |
| 1 | 2 | Ko1 | 2.2.1 proper | 27.79 | Neg | 29.32 | 28.34 |
| 2 | 2 | Ko2 | 2.2.1 proper | 19.72 | Neg | 20.47 | 19.07 |
| 3 | 2 | Ko3 | 2.2.1 proper | 26.28 | Neg | 26.13 | 24.74 |
| 4 | 2 | Ko4 | 2.2.1 proper | 27.52 | Neg | 29.26 | 28.22 |
| 5 | 2 | Ko5 | 2.2.1 proper | 27.99 | Neg | 29.63 | 28 |
| 6 | 2 | Ko6 | 2.2.1 proper | 25.37 | Neg | 26.07 | 24 |
| 7 | 2 | Ko7 | 2.2.1 proper | 23.3 | Neg | 24.2 | 22.56 |
| 8 | 2 | Ko8 | 2.2.1 proper | 20.58 | Neg | 22.76 | 21.64 |
| 9 | 2 | 351 | 2.2.1 proper | 36.47 | Neg | 36.29 | 37.16 |
| 10 | 2 | 352 | 2.2.1 proper | 36.53 | Neg | 36.58 | 36.74 |
| 11 | 2 | 353 | 2.2.1 proper | 25.97 | Neg | 26.25 | 26.22 |
| 12 | 2 | 354 | 2.2.1 proper | 32.47 | Neg | 32.83 | 33.22 |
| 13 | 2 | 355 | 2.2.1 proper | 31.43 | Neg | 32.03 | 33.26 |
| 14 | 2 | 356 | 2.2.1 proper | 34.04 | Neg | 34.92 | 33.96 |
| 15 | 2 | 357 | 2.2.1 proper | 38.83 | 39.03 | 36.36 | 37.33 |
| 16 | 2 | 358 | 2.2.1 proper | 31.85 | Neg | 30.9 | 36.99 |
| 17 | 2 | 359 | 2.2.1 proper | 29.23 | Neg | 29.84 | 29.72 |
| 18 | 2 | 360 | 2.2.1 proper | 27.88 | Neg | 28.26 | 27.8 |
| 19 | 2 | 361 | 2.2.1 proper | 31.53 | Neg | 32.18 | 30.41 |
| 20 | 7 | 351 | 2.2.1 proper | 38.18 | Neg | 36.79 | Neg |
| 21 | 7 | 352 | 2.2.1 proper | 39.31 | Neg | 35.9 | Neg |
| 22 | 7 | 354 | 2.2.1 proper | Neg | Neg | 38.48 | Neg |
| 23 | 7 | 355 | 2.2.1 proper | 37.57 | Neg | 37.13 | Neg |
| 24 | 7 | 356 | 2.2.1 proper | 38 | Neg | 35.96 | 34.54 |
| 25 | 7 | 357 | 2.2.1 proper | 33.81 | Neg | 33.81 | Neg |
| 26 | 7 | 358 | 2.2.1 proper | 37.36 | Neg | 35.61 | Neg |
| 27 | 7 | 359 | 2.2.1 proper | 38.38 | Neg | 36.93 | Neg |
| 28 | 7 | 360 | 2.2.1 proper | 38.77 | Neg | 35.56 | Neg |
| 29 | 7 | 361 | 2.2.1 proper | 38.22 | Neg | 36.39 | 38.92 |
| 30 | 2 | Ko1 | 2.2.1 variant | Neg | 23.59 | 23.37 | 24.01 |
| 31 | 2 | Ko2 | 2.2.1 variant | Neg | 25.55 | 25.89 | 27.35 |
| 32 | 2 | Ko3 | 2.2.1 variant | Neg | 26.3 | 25.23 | 26.67 |
| 33 | 2 | Ko4 | 2.2.1 variant | Neg | 27.83 | 28.1 | 29.31 |
| 34 | 2 | Ko5 | 2.2.1 variant | Neg | 22.9 | 22.8 | 23.24 |
| 35 | 2 | Ko6 | 2.2.1 variant | Neg | 29.06 | 28.97 | 30.97 |
| 36 | 2 | Ko7 | 2.2.1 variant | Neg | 28.46 | 28.19 | 28.56 |
| 37 | 2 | Ko8 | 2.2.1 variant | Neg | 27.81 | 26.44 | 28.9 |
| 38 | 2 | Ko10 | 2.2.1 variant | Neg | 25.55 | 25.83 | 26.66 |
| 39 | 2 | Ko11 | 2.2.1 variant | Neg | 23.04 | 22.92 | 23.53 |
| 40 | 2 | 288 R | 2.2.1 variant | Neg | Neg | Neg | Neg |
| 41 | 2 | 251 R | 2.2.1 variant | Neg | Neg | Neg | Neg |
| 42 | 2 | 285 R | 2.2.1 variant | Neg | Neg | Neg | Neg |
| 43 | 2 | 271 R | 2.2.1 variant | Neg | Neg | Neg | Neg |
| 44 | 2 | 272 R | 2.2.1 variant | Neg | Neg | Neg | Neg |
| 45 | 2 | 266 R | 2.2.1 variant | Neg | Neg | Neg | Neg |
| 46 | 2 | 261 R | 2.2.1 variant | Neg | Neg | Neg | Neg |
| 47 | 2 | 290 R | 2.2.1 variant | Neg | Neg | Neg | Neg |
| 48 | 2 | 260 R | 2.2.1 variant | Neg | Neg | Neg | Neg |
| 49 | 2 | 287 R | 2.2.1 variant | Neg | Neg | Neg | Neg |
| 50 | 2 | 262 R | 2.2.1 variant | Neg | Neg | Neg | Neg |
| 51 | 2 | 252 R | 2.2.1 variant | Neg | Neg | Neg | Neg |
| 52 | 2 | 5 | 2.2.1 variant | Neg | 35.62 | 35.84 | 37.83 |
| 53 | 2 | 336 | 2.2.1 variant | Neg | 30.12 | 30.5 | 32.18 |
| 54 | 2 | 331 | 2.2.1 variant | Neg | 33.48 | 33.75 | 36.06 |
| 55 | 2 | 339 | 2.2.1 variant | Neg | 31.16 | 31.03 | 33.05 |
| 56 | 2 | 316 | 2.2.1 variant | Neg | 29.99 | 29.1 | 30.12 |
| 57 | 2 | 329 | 2.2.1 variant | Neg | 29.43 | 28.6 | 29.5 |
| 58 | 2 | 327 | 2.2.1 variant | Neg | 37.99 | 36.94 | Neg |
| 59 | 2 | 311 | 2.2.1 variant | Neg | 35.94 | 34.72 | 37.56 |
| 60 | 2 | 350 | 2.2.1 variant | Neg | Neg | Neg | 36.32 |
| 61 | 2 | 343 | 2.2.1 variant | Neg | 29.79 | 30.2 | 31.12 |
| 62 | 2 | 326 | 2.2.1 variant | Neg | Neg | 37.23 | Neg |
| 63 | 2 | 328 | 2.2.1 variant | Neg | 32.97 | 34 | 35.81 |
