# Supplementary material for: Simultaneous detection and differentiation by multiplex real time RT-PCR of highly pathogenic avian influenza subtype H5N1 classic (clade 2.2.1 proper) and escape mutant (clade 2.2.1 variant) lineages in Egypt
Source: Virol J. 2010 Oct 7;7:260. doi: 10.1186/1743-422X-7-260 (PMC2958913; doi:10.1186/1743-422X-7-260)
Supplement: Additional file 4 — Detection and differentiation of HPAIV H5N1 in pooled swab samples collected from commercial poultry and backyard birds in Egypt in 2008- 2010 by multiplex H5 RT-qPCR compared to standard generic H5 and M gene RT-qPCR protocols. [file 1743-422X-7-260-S4.DOC]

**Table S4.** Detection and differentiation of HPAIV H5N1 in pooled swab samples collected from commercial poultry and backyard birds in Egypt in 2008- 2010 by multiplex H5 RT-qPCR compared to standard generic H5 and M gene RT-qPCR protocols.

| **No.** | **Host** | **Origin** | **Vaccination status** | **Multiplex RT-qPCR assay** | | | **Generic RT-qPCR H5** | **Generic RT-qPCR M** |
| --- | --- | --- | --- | --- | --- | --- | --- | --- |
|  |  |  |  | **2.2.1 proper HEX** | **2.2.1 variant FAM** | **2.2.1**  **all**  **ROX** |  |  |
| 1 | Chicken | Backyard | unvaccinated | Neg | 16.02 | 17.23 | 27.09 | 21.7 |
| 2 | Chicken | Backyard | unvaccinated | Neg | 19.08 | 20.17 | 30.22 | 29.77 |
| 3 | Chicken | Backyard | unknown | Neg | 20.06 | 20.3 | 26.25 | 35.94 |
| 4 | Chicken | Farm | vaccinated | Neg | 20.08 | 21.01 | 28.33 | 30.87 |
| 5 | Chicken | Farm | vaccinated | Neg | 20.91 | 21.34 | 26.99 | 26.22 |
| 6 | Chicken | Backyard | unvaccinated | Neg | 20.99 | 21.39 | 25.33 | 27.51 |
| 7 | Chicken | Backyard | unvaccinated | Neg | 22.09 | 23.09 | 26.15 | 28.69 |
| 8 | Chicken | Farm | vaccinated | Neg | 22.14 | 22.39 | 32.43 | 33.02 |
| 9 | Chicken | Backyard | unvaccinated | 23.55 | 22.68 | 23.92 | 27.04 | 30.84 |
| 10 | Chicken | Farm | vaccinated | Neg | 23.7 | 24.79 | Neg | 29.65 |
| 11 | Chicken | Farm | vaccinated | Neg | 23.88 | 24.01 | 22.08 | 24.25 |
| 12 | Chicken | Backyard | unknown | Neg | 26.07 | 27.03 | 29.36 | 29.57 |
| 13 | Chicken | Farm | vaccinated | Neg | 26.77 | 27.17 | 34.72 | 31.78 |
| 14 | Turkey | Farm | unknown | Neg | 26.85 | 27.23 | 31.74 | 32.95 |
| 15 | Chicken | Farm | vaccinated | Neg | 27.21 | 28.19 | 31.09 | 30.34 |
| 16 | Chicken, duck | Backyard | unknown | Neg | 29.96 | 30.97 | 30.9 | 32.87 |
| 17 | Chicken | Farm | vaccinated | 33.69 | 34.94 | 32.76 | Neg | 27.11 |
| 18 | Chicken | Farm | vaccinated | 36.61 | 36.38 | 37.11 | Neg | 36.69 |
| 19 | Chicken | Backyard | unknown | 28.79 | Neg | 30.21 | 30.9 | 36.46 |
| 20 | Duck | Backyard | vaccinated | 19.87 | Neg | 21.32 | 22.59 | 28.77 |
| 21 | Duck | Backyard | vaccinated | 17.94 | Neg | 19.29 | 22.53 | 27.46 |
| 22 | duck | Backyard | unvaccinated | 21.79 | Neg | 23.27 | 26.95 | 31.37 |
| 23 | Chicken | Backyard | unvaccinated | 24.79 | Neg | 26.26 | 37 | 32.37 |
| 24 | Duck | Backyard | unvaccinated | 21.85 | Neg | 24.82 | 26.07 | 28.87 |
| 25 | Chicken | Backyard | unvaccinated | 21.92 | Neg | 23.88 | 28.53 | 33.61 |
| 26 | Goose | Backyard | unknown | 21.87 | Neg | 23.27 | 28.6 | 28.95 |
| 27 | Chicken | Farm | vaccinated | 29.9 | Neg | 22.21 | 27.43 | 35.35 |
| 28 | Chicken | Backyard | unknown | 18.84 | Neg | 20.24 | 21.76 | 26.41 |
| 29 | Duck | Backyard | unvaccinated | 23.8 | Neg | 24.98 | 30.86 | 37.08 |
| 30 | Chicken | Backyard | vaccinated | 17.6 | Neg | 18.92 | 30.14 | 29.00 |
| 31 | Duck | Backyard | unvaccinated | 26.68 | Neg | 27.93 | Neg | 32.96 |
| 32 | Duck | Backyard | unvaccinated | 20.73 | Neg | 21.94 | Neg | 27.3 |
| 33 | Chicken | Backyard | vaccinated | 16.75 | Neg | 18.07 | 26.08 | 25.84 |
| 34 | Duck | Backyard | unvaccinated | 22.63 | Neg | 23.12 | 30.83 | 32.78 |
| 35 | Duck | Backyard | unvaccinated | 25.65 | Neg | 26.04 | 31.24 | 30.25 |
| 36 | Duck | Backyard | unvaccinated | 19.84 | Neg | 21.18 | 27.58 | 28.45 |
| 37 | Chicken | Backyard | unvaccinated | 16.6 | Neg | 17.83 | 21.77 | 20.86 |
| 38 | Chicken | Backyard | unvaccinated | 17.62 | Neg | 18.74 | 24.06 | 27 |
| 39 | Duck | Backyard | unknown | 22.55 | Neg | 23.88 | 26.67 | 26.66 |
| 40 | Chicken, duck,  goose | Backyard | vaccinated | 19.75 | Neg | 20.12 | 20.02 | 22.84 |
| 41 | Chicken | Backyard | unvaccinated | 18.76 | Neg | 20.12 | 28.32 | 26.45 |
| 42 | Chicken | Backyard | unknown | 15.67 | Neg | 17 | 24.34 | 25.32 |
| 43 | Chicken | Backyard | unknown | 15.12 | Neg | 16.84 | 22.92 | 24.5 |
| 44 | Goose | Backyard | unvaccinated | 24.01 | Neg | 25.37 | 28.73 | 30.39 |
| 45 | Goose | Backyard | unknown | 27.75 | Neg | 28.96 | 33.39 | 35.01 |
| 46 | Duck | Backyard | unvaccinated | 26.64 | Neg | 27.88 | 29.84 | 37.03 |
| 47 | Duck | Backyard | unvaccinated | 21.93 | Neg | 24.7 | 24.19 | 32.26 |
| 48 | Chicken | Backyard | unvaccinated | 23.72 | Neg | 24.08 | 29.16 | 30.75 |
| 49 | Chicken, duck | Backyard | unvaccinated | 23.94 | Neg | 25.23 | 36.47 | 34.24 |
| 50 | Duck | Backyard | unvaccinated | 26.67 | Neg | 27.02 | 28.62 | 28.22 |
